# Artificial Intelligence and Male Infertility

**DOI:** 10.5935/1518-0557.20250024

**Published:** 2025

**Authors:** Paulo Franco Taitson

**Affiliations:** Professor, Pontifical Catholic University of Minas Gerais (PUC Minas), Assistant Editor, JBRA Assisted Reproduction

Infertility is defined as the inability to conceive after 1 year of unprotected intercourse. Male infertility is a disease, affecting millions of men around the world. Several factors, such as lifestyle, genetics, and environmental conditions, contribute to the reduction of semen quality and reproductive capacity. There exist substantial data to suggest a decline in sperm counts over time. Although causative factors have yet to be fully elucidated, potential causes include, increased rates of obesity, poor diet, and exposure to environmental toxins. In this context, artificial intelligence (AI) has become an innovative tool, bringing significant advances that can help in the diagnosis, treatment, psycho-emotional repercussions and research on infertility.

AI methods have shown to be a promising tool in the field of health. Recent work has demonstrated that these methods can develop effective diagnostic, treatments and predictive tools to identify various diseases. In the future, AI-based programs may become an integral part of patients’ hospital visits with their ability to assist in diagnosis and management of reproductive diseases. Scientists and physicians should take an active approach to understand the theories behind AI and its utility in health with the goal of providing optimal patient care (Calogero *et al*., 2024; Fang *et al*., 2025).

With AI, algorithms trained on large databases can analyze microscopic patterns in sperm, assessing their morphology, motility, and concentration with high accuracy. This allows, combined with the observation of an examiner trained in seminal analysis, faster and more reliable diagnoses, enabling more targeted treatments. An example of this is in a study published in Scientific Reports in 2024, where Kobayashi *et al*. (2024) decided to develop an AI model that, according to them, predicts the risk of male infertility based on the hormone levels present in a blood test. The study was based on data from 3,662 patients and had an accuracy rate of approximately 74%. In particular, the tool obtained better results in predicting the risk of non-obstructive azoospermia. The same author, last year, already highlighted the horizon of application of AI in male infertility in a publication in Reproductive Medicine and Biology.

Ghayda *et al*. (2024) published an article in the World Journal Men’s Health, where the authors highlight how AI-based automated predictions can offer consistency and efficiency in terms of time and cost in infertility research and clinical management. Despite all these advances, the use of AI in male infertility still faces challenges. Ethical issues, data privacy, and the need for regulation are aspects that need to be considered to ensure the safe and effective use of this technology.

Multiple studies have utilized Artificial Intelligence (AI) to analyze clinical-reproductive characteristics, potential urology, varicocele management and semen analyses. These AI-driven approaches aim to discover novel biomarkers that can assess, stratify, and prognosticate men infertile. These sophisticated methodologies offer new insights and strategies for understanding normal spermatogenesis and the pathophysiology of male infertility. The application of AI strategies is expected to revolutionize male health management, enhancing male fertility and optimizing reproductive outcomes.

Looking ahead, the future of AI in male infertility analysis will be shaped by ethical and accessible integration. AI-guided surgical robots that monitor signals in real time and virtual assistants that guide patients at home are just the beginning. Artificial intelligence is profoundly transforming reproductive science, promising a future where more accurate diagnoses, personalized treatments, and more efficient systems become the norm. In a horizon that has already begun to take shape in 2023, AI is not only an auxiliary tool, but an essential partner for doctors, patients, and managers, redefining the limits of health care.
